# Partisans neither expect nor receive reputational rewards for sharing falsehoods over truth online

**DOI:** 10.1093/pnasnexus/pgae287

**Published:** 2024-07-24

**Authors:** Isaias Ghezae, Jillian J Jordan, Izzy B Gainsburg, Mohsen Mosleh, Gordon Pennycook, Robb Willer, David G Rand

**Affiliations:** Department of Sociology, Stanford University, Stanford, CA 94305, USA; Negotiation, Organizations and Markets Unit, Harvard Business School, Boston, MA 02163, USA; Department of Sociology, Stanford University, Stanford, CA 94305, USA; Management Department, University of Exeter Business School, Exeter EX4 4PU, UK; Sloan School of Management, Massachusetts Institute of Technology, Cambridge, MA 02142, USA; Department of Psychology, Cornell University, Ithaca, NY 14850, USA; Department of Sociology, Stanford University, Stanford, CA 94305, USA; Sloan School of Management, Massachusetts Institute of Technology, Cambridge, MA 02142, USA; Institute for Data, Systems, and Society, Massachusetts Institute of Technology, Cambridge, MA 02142, USA; Department of Brain and Cognitive Sciences, Massachusetts Institute of Technology, Cambridge, MA 02142, USA

**Keywords:** partisanships, signaling, fake news, misinformation, social media

## Abstract

A frequently invoked explanation for the sharing of false over true political information is that partisans are motivated by their reputations. In particular, it is often argued that by indiscriminately sharing news that is favorable to one's political party, regardless of whether it is true—or perhaps especially when it is *not* true—partisans can signal loyalty to their group, and improve their reputations in the eyes of their online networks. Across three survey studies (total *N* = 3,038), and an analysis of over 26,000 tweets, we explored these hypotheses by measuring the reputational benefits that people anticipate and receive from sharing different content online. In the survey studies, we showed participants actual news headlines that varied in (ⅰ) veracity, and (ⅱ) favorability to their preferred political party. Across all three studies, participants anticipated that sharing true news would bring more reputational benefits than sharing false news. Critically, while participants also expected greater reputational benefits for sharing news favorable to their party, the perceived reputation value of veracity was no smaller for more favorable headlines. We found a similar pattern when analyzing engagement on Twitter: among headlines that were politically favorable to a user's preferred party, true headlines elicited more approval than false headlines.

## Introduction

The spread of political misinformation online has become a topic of great societal concern and scholarly research in recent years (1, 2). One key research focus has been explaining why people share false versus true information—an agenda that is both scientifically valuable and critical for developing effective interventions to reduce the spread of misinformation.

One influential line of theorizing has emphasized the role of social and group motivations in the sharing of false news relative to true news online (3–6). This research has focused on the role of political partisanship, arguing that partisans are often willing to share content that aligns with (i.e. is favorable to) the beliefs and preferences of their political ingroup, regardless of its accuracy—or even *because* of its *inaccuracy*, insofar as sharing *false* partisan news is a particularly effective way to signal one's partisan commitment (5, 6).

Building on this line of reasoning, many pundits and members of the general public, as well as scholars across a diverse range of fields including political science (5), psychology (7), business (8), and philosophy (9), have argued that reputational concerns, and the desire for social rewards from co-partisans, play an important role in driving the sharing of false news over true news.

There is a surprising lack, however, of direct evidence evaluating this frequently invoked claim. It stands to reason that *if* people anticipate being socially rewarded for indiscriminately sharing partisan news, reputation motives may increase their tendency to do so (8, 10). Yet, it remains unclear whether people actually anticipate or receive greater social approval for sharing politically-favorable news without regard—or even with *negative* regard—for its veracity. Here, we investigate this issue empirically.

Across a series of survey studies (Study 1, *N* = 1,319; Study 2, *N* = 853; Study 3, *N* = 866), we presented participants with various news headlines (total *k* = 588 headlines), asking them to report the reputational gains they would expect to receive from members of their social network if they were to share each news item. We also asked them to assess the extent to which each news item was (ⅰ) true or false (veracity), and (ⅱ) favorable to their political party or not (favorability). We then examined the relationship between the perceived accuracy and favorability of headlines and the anticipated reputational returns from sharing that news item, allowing us to directly test whether participants anticipate being socially rewarded for sharing news they think is inaccurate versus accurate, and how the perceived reputation value of veracity varies as a function of a headline's partisan favorability. Alongside participants’ perceived accuracy ratings, we also provide a more objective measure of veracity by categorizing headlines as “false” if we collected them from fact-checking outlets (that classified them as false), and “true” if we collected them from reputable mainstream outlets.

Finally, we complement our analysis of participants’ perceptions of the reputation value of sharing different content with an analysis of the *actual* social approval users received when sharing true and false claims on Twitter. To achieve this, we examine the same set of news items rated by participants in our surveys and measure how much approval (using a validated metric from Frimer et al. (11)) Twitter users received when sharing each news item.

## Results

**Fig. 1.**
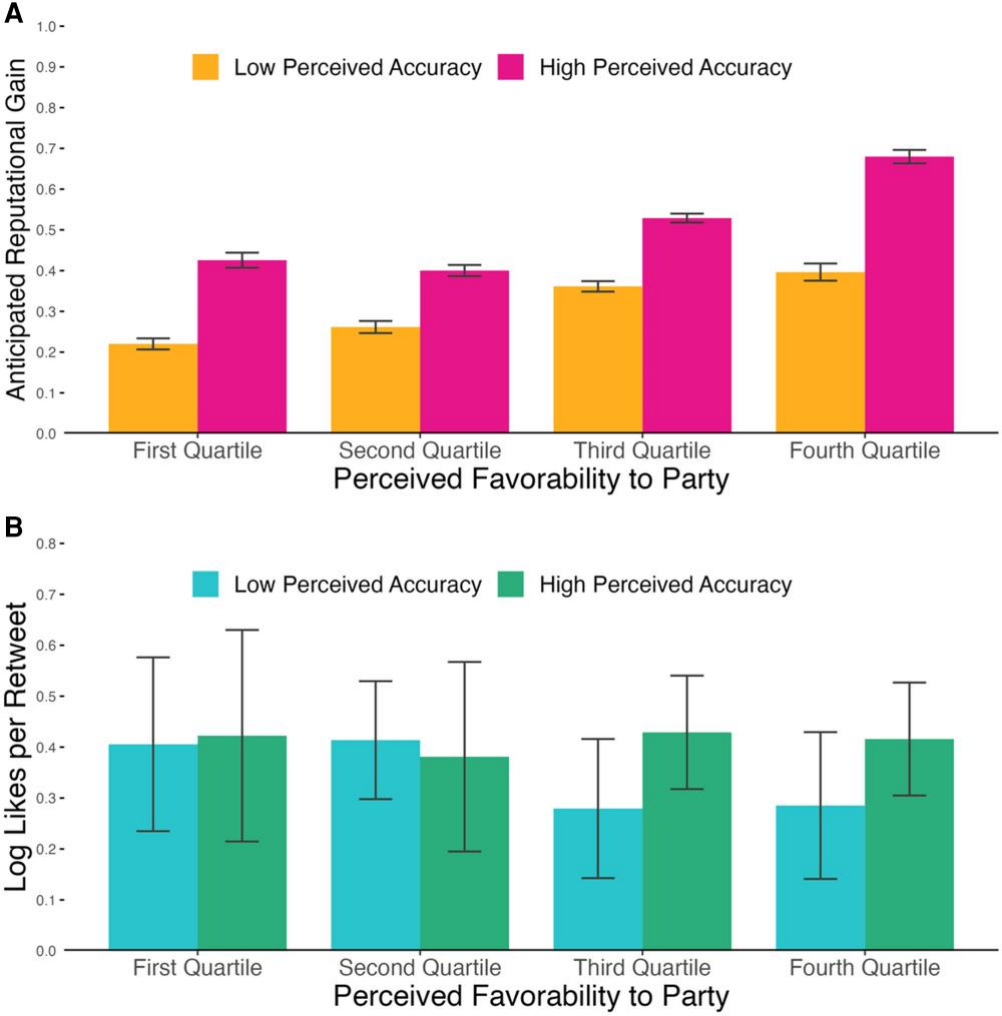
A) Anticipated reputational benefits for sharing a headline as a function of its perceived accuracy (median split) and favorability (quartile split) to the participant's party (pooled across the three surveys). B) Approval received for sharing a headline on Twitter, as measured by log likes-per-retweet, as a function of its averaged perceived accuracy (median split) and favorability (quartile split). Error bars represent 95% CIs.

Across all three studies, we do not find that participants anticipate reputational benefits from indiscriminately sharing favorable news regardless of its veracity, or from preferentially sharing misinformation relative to accurate news. To the contrary, as shown in Fig. 1A, we found a *positive* relationship between perceived (i.e. participant-rated) accuracy and anticipated reputational gains from sharing (S1: *b* = 0.694, S2: *b* = 0.665, S3: *b* = 0.821; all *P*s < 0.001). There was also a positive relationship between a headline's political favorability and anticipated reputational gains (S1: *b* = 0.702, S2: *b* = 0.347, S3: *b* = 0.354; all *P*s < 0.001). And critically, we found a significant positive interaction between perceived accuracy and favorability (S1: *b* = 0.087, S2: *b* = 0.139, S3: *b* = 0.095; all *P*s < 0.001), reflecting that the perceived reputation value of accuracy was greater for more favorable headlines.

For all three studies, the main effects of accuracy and favorability were robust to controlling for other perceived traits of the headline, like familiarity or importance, the interactions between these traits and accuracy, and the interactions between these traits and favorability. However, including these controls rendered the interaction between accuracy and favorability nonsignificant—but, importantly, did not cause it to reverse (for details, see Extended Results, available at ref. (12)). Thus, even in models with controls, we find no evidence that the perceived reputation value of veracity becomes less positive for politically favorable headlines.

We also observe similar results in models that replace a headline's perceived accuracy with its *objective* veracity (positive association between objective veracity and reputation value: S1: *b* = 0.340, S2: *b* = 0.168, S3: *b* = 0.194, all *P*s < 0.001; positive association between favorability and reputation value: S1: *b* = 0.836, S2: *b* = 0.524, S3: *b* = 0.563, all *P*s < 0.001; no significant interactions between objective veracity and favorability on reputation value: S1: *b* = 0.021, *P* = 0.413; S2: *b* = −0.020, *P* = 0.331; S3: *b* = −0.015, *P =* 0.504). This analysis suggests that our perceived accuracy findings do not merely reflect that (i) people preferentially believe false favorable content, or (ii) social desirability concerns discourage participants from rating false content as reputationally favorable.

Because the sharing of fake news is relatively uncommon among the general population, we also conduct a robustness check to investigate the participants who were the most inclined to share fake news. To this end, we categorized participants as “high fake news sharers” if, for over half of the objectively false headlines they were shown, they provided an above-midpoint rating of their likelihood of sharing the headline. This approach classifies a relatively small number of participants as high fake news sharers (*N* = 637 across our three studies, corresponding to 21% of our total sample), so we pool data across our three studies for this robustness check. Among high fake news sharers, both perceived accuracy and political favorability are positively associated with anticipated reputational gain (perceived accuracy: *b* = 0.128, *P* < 0.001; favorability: *b* = 0.057, *P* < 0.001), and we find no significant interaction between perceived accuracy and political favorability on anticipated reputational gain (*b* = 0.006, *P* = 0.217). When considering objective veracity, we find no significant association between objective veracity and anticipated reputational gain (*b* = 0.006, *P* = 0.168), a positive association between political favorability and anticipated reputational gain (*b* = 0.088, *P* < 0.001), and no significant interaction between objective veracity and political favorability (*b* = −0.002, *P* = 0.648). We find similar results when adding to our models the one control variable (ratings of a headline's importance) that was measured in all three studies, and thus can be included in our pooled analysis. For further details about this robustness check, see Extended Results (12).

We also conducted a robustness check investigating strongly committed partisans, who might be the most interested in obtaining social approval from co-partisans. Among such participants, when considering perceived accuracy, we find positive associations between perceived accuracy and anticipated reputational gain (S1: *b* = 0.803, S2: *b* = 0.828, S3: *b* = 1.046; all *P*s < 0.001), positive associations between political favorability and anticipated reputational gain (S1: *b* = 0.808, S2: *b* = 0.442, S3: *b* = 0.449; all *P*s < 0.001), and positive interactions between perceived accuracy and favorability (S1: *b* = 0.087, S2: *b* = 0.137, S3: *b* = 0.152; all *P*s < 0.001). When considering objective veracity, we find positive associations between objective veracity and anticipated reputational gain (S1: *b* = 0.394, S2: *b* = 0.157, S3: *b* = 0.157, all *P*s < 0.001), positive associations between favorability and anticipated reputational gain (S1: *b* = 1.008, S2: *b* = 0.686, S3: *b* = 0.758, all *P*s < 0.001), and no interaction between objective veracity and favorability in Studies 1 or 2 (S1: *b* = 0.039, *P* = 0.242; S2: *b* = −0.030, *P* = 0.240), although we do find a negative interaction in Study 3 (*b* = −0.070, *P* = 0.009). Furthermore, we find similar results when adding control variables to our models. Additional details about this robustness check can be found in Extended Results (12).

Our Extended Results also reveal that our main results replicate when analyzing a secondary reputation variable, in which participants were asked about the extent to which sharing a headline would make them appear like a strong and loyal partisan (12). Overall, then, our set of robustness checks provides further evidence that participants—even those who are relatively more likely to share fake news, or relatively stronger partisans—anticipate positive reputation consequences from sharing true (vs. false) headlines, and casts further doubt on the hypothesis that the perceived reputation value of veracity is weaker for politically favorable headlines.

Finally, we turn to instances where the same headlines were shared on Twitter and evaluate how accuracy and favorability relate to the approval they receive, in order to illuminate what kinds of news content Twitter users *actually* approve of. For each headline featured in our survey experiments, to measure approval of tweets sharing the headline, we calculate the average logged likes-per-retweet across such tweets [an approach validated by Frimer et al. (11)]. In our primary analyses, we exclude from our calculation instances in which users tweeted politically incongruent headlines (i.e. Democrats tweeted Republican-leaning headlines, or vice versa), given that such tweets might reflect criticism rather than endorsement of the relevant headline. Then, using the ratings from the surveys, we computed the average veracity and political favorability of each headline (SI Appendix for full methods).

Consistent with survey respondents’ expectations, we find no evidence that, when headlines are favorable, Twitter users approve of disregarding veracity (Fig. 1B). Veracity was not significantly associated with approval when considering perceived accuracy (*b* = 0.021, *P* = 0.444) but was significantly positively associated with approval when considering objective veracity (*b* = 0.075, *P* = 0.019). Favorability was not significantly associated with approval, either in models that consider perceived accuracy (*b* = −0.022, *P* = 0.429) or objective veracity (*b* = −0.055, *P* = 0.069). Additionally, we found a marginally significant positive interaction between favorability and perceived accuracy (*b* = 0.049, *P* = 0.056), as well as a significant positive interaction between favorability and objective veracity (*b* = 0.066, *P* = 0.031).

Finally, in a robustness check that *does* analyze instances in which users tweeted politically incongruent headlines, we find that perceived accuracy and objective veracity are both positively associated with social approval, while favorability is not, and find no significant interactions between either veracity measure and favorability on approval [for more detail, see Extended Results (12)].

## Discussion

We consistently failed to find evidence that reputational concerns can motivate people to preferentially share falsehoods over truth, or indiscriminately share politically favorable content without regard for the truth. While participants in our surveys anticipated reputational benefits from sharing politically favorable news, they also expected to gain reputationally from sharing *accurate* information. And critically, the perceived reputation value of veracity did not attenuate or reverse for headlines that were more politically favorable. As such, participants anticipated the greatest reputational benefits for sharing news that was both politically favorable *and* true. Moreover, when examining actual approval that Twitter users received when sharing the same news headlines, we found evidence that sharing true news elicited more approval, and that this association was no less positive for politically favorable headlines. Thus, although recent research has pointed to the possibility that people might be particularly inclined to share politically favorable misinformation to gain reputational advantages (5, 7, 9), our findings do not support this hypothesis. Instead, we find evidence that partisans accurately anticipate that attending to the accuracy of politically aligned information—and preferentially sharing *true* favorable news—is the better way to win social approval.

Our results thus raise the question of what *does* contribute to the preferential sharing of false versus true news, and suggest that factors other than reputation motives—such as limits to people's ability to distinguish false from true news, or inattention to the veracity of news when making sharing decisions (13, 14)—may be key drivers of undiscerning sharing of information.

Importantly, while our results suggest that reputation motives are unlikely to encourage the preferential sharing of false relative to true news, they do not rule out the possibility that when people share misinformation, reputation motives contributed to their sharing decisions. Indeed, reputation motives may generally be an important driver of information sharing on social media, such that whenever an individual shares a headline, she tends to (implicitly or explicitly) anticipate that sharing will make her look good—including in cases where the headline was false. Critically, however, we do not find evidence that reputation motives systematically encourage people to share false relative to true information, or to disregard the veracity of headlines that favor their political parties. As such, our work casts doubt on the hypothesis that reputation motives are to blame for undiscerning sharing behavior.

## Materials and methods

### Surveys

Studies 1, 2, and 3 were conducted in January 2021, November 2021, and April 2022, respectively. Participants were recruited via Amazon Mechanical Turk for Study 1 and Lucid for Studies 2 and 3 [for demographic details about our samples, see Extended Methods (12)]. We only included participants who passed our attention checks. For our survey analyses, we shaped our data to one observation per headline per participant, then used linear regressions with robust standard errors clustered by participant and headline.

Research was approved by the Stanford University and University of Regina Institutional Boards. All subjects provided informed consent (SI Appendix for detailed methods of the surveys).

### Twitter analysis

See SI Appendix for full methods of the Twitter analysis.

## Supplementary Material

pgae287_Supplementary_Data

## Data Availability

Data and analysis code files have been deposited in the Open Science Framework (https://osf.io/5jwgd/).

## Preprints

This manuscript was posted on a preprint: https://doi.org/10.31219/osf.io/qjgdr.
