# Supplementary material for: Partisans neither expect nor receive reputational rewards for sharing falsehoods over truth online
Source: PNAS Nexus. 2024 Jul 24;3(8):pgae287. doi: 10.1093/pnasnexus/pgae287 (PMC11348091; doi:10.1093/pnasnexus/pgae287)
Supplement: pgae287_Supplementary_Data [file pgae287_supplementary_data.docx]

**Supporting information for “Partisans neither expect nor receive reputational rewards for sharing falsehoods over truth online”**

Isaias Ghezae^*^, Jillian J. Jordan^*^, Izzy B. Gainsburg, Mohsen Mosleh, Gordon Pennycook, Robb Willer, David G. Rand

*Indicates equal contribution

^ǂ^Corresponding authors: Isaias Ghezae ([ighezae@stanford.edu](mailto:ighezae@stanford.edu)); Jillian J. Jordan ([jijordan@hbs.edu](mailto:jijordan@hbs.edu))

**This PDF file includes:** Supporting text

SI References

**Supporting Information Text**

**Extended Methods for surveys**

**Study 1 Procedure (not preregistered)**

Study 1 was our only non-preregistered study and was conducted in January 2021. Participants provided informed consent and were then given an attention check immediately after providing consent. For the attention check, participants were asked “Puppy is to dog as kitten is to ?”, with the word “cat” being the correct answer. Participants who did not pass the first attention check were not allowed to complete the survey. We only conducted analyses with participants who passed the two attention checks in each study, unless otherwise noted. Participants were then asked to indicate their party affiliation (i.e., either being a Democrat, Republican, or independent) and were asked to indicate if they were a strong Democrat or Republican. For participants who indicated that they were an independent, they were asked if they leaned closer to either of the political parties or if they were a true independent. We classified independents as Democrats or Republicans if they leaned toward one of the parties. Additionally, true independents were asked, if they were forced to pick a political party, which party do they identify more closely with, and were classified as Democrats or Republicans based on their response.

Participants were then told that they would be presented with a series of actual news headlines and that they would answer a series of questions for each headline. Participants were then shown 10 headlines that were randomly sampled from a total set of 140 headlines. Each headline was shown one at a time and participants were asked questions related to one headline before being shown the next headline. Included in these questions were our main independent variables (political favorability and perceived accuracy) and our main dependent variable (anticipated reputational gain). All questions were presented in a random order except the final question asking participants how likely they would be to share the headline on social media.

Participants were asked to rate the perceived accuracy of the headline. In all studies, perceived accuracy was measured using the following question: “What is the likelihood that the above headline is true?” In Study 1, participants used a 9-pt scale to answer this question (1 = not at all likely; 5 (midpoint) = somewhat likely; 9 = very likely).

Participants were also asked about the political favorability of the headline. Political favorability was measured using the following question: "Assuming the above headline is entirely accurate, how favorable would it be to Democrats versus Republicans?" In Study 1, participants used a 9-pt scale to answer this question (1 = very favorable for Democrats; 5 (midpoint) = neutral; 9 = very favorable for Republicans). This question was recoded with respect to the participant’s party such that a higher score means higher favorability to the participant’s own party (i.e., reverse coded for Democrats).

Participants were asked how sharing the headline would benefit Democrats or Republicans: “If you were to share the above headline on social media, how much would this benefit Democrats versus Republicans?” Participants used a 9-pt scale to answer this question (1 = strongly benefit Democrats; 5 (midpoint) = neutral; 9 = strongly benefit Republicans).

Participants were asked how important the headline is: “Assuming the headline is entirely accurate, how important would this news be?” Participants used a 9-pt scale to answer this question (1 = not at all important; 5 (midpoint) = somewhat important; 9 = very important).

Participants were asked how funny the headline is: “How funny is this headline?” Participants used a 9-pt scale to answer this question (1 = not at all funny; 5 (midpoint) = somewhat funny; 9 = very funny).

Participants were asked how surprising the headline is: “How surprising is this headline?” Participants used a 9-pt scale to answer this question (1 = not at all surprising; 5 (midpoint) = somewhat surprising; 9 = very surprising).

Participants were asked how sharing the headline would make them seem like a strong and loyal partisan: “If you were to share this headline on social media (such as Facebook, Twitter, or Instagram), using your own account, how much would people in your social network see you as a strong and loyal [Democrat]/[Republican]?” Participants used a 9-pt scale to answer this question (1 = not at all; 5 (midpoint) = somewhat; 9 = very).

Participants were asked how likely people would engage with the headline if they shared it: “If you were to share this headline on social media (such as Facebook, Twitter, or Instagram), using your own account, how likely would people in your social network be to engage with your post (e.g., by liking it, commenting on it, or sharing it)?” Participants used a 9-pt scale to answer this question (1 = not at all; 5 (midpoint) = somewhat; 9 = very).

Participants answered our key dependent variable, anticipated reputational gain, which was measured using the following question: “If you were to share this headline on social media (such as Facebook, Twitter, or Instagram), using your own account, how positively would you be viewed, overall, by people in your social network?” Participants used a 9-pt scale to answer this question (1 = not at all; 5 (midpoint) = somewhat; 9 = very).

Finally, participants were asked how likely they would share the headline themselves if they were to encounter it on social media: “If you were to see the above headline on social media, how likely would you be to share it?” Participants used a 9-pt scale to answer this question (1 = not at all likely to share; 5 (midpoint) = somewhat likely to share; 9 = very likely to share). While the other questions were presented in a random order, this question was always asked last for each headline.

After answering the questions for all ten headlines, participants were then given a second attention check. Specifically, participants were presented with a paragraph of text that ultimately asked them to indicate their favorite colors. In the middle of the paragraph, participants were told to ignore the instructions and indicate “red” and “green” as their favorite colors.

Finally, participants were asked the following demographic and exploratory questions: age, gender, education, income, ethnicity, political ideology, 2020 presidential vote choice, 018 congressional vote choice, favorability towards Donald Trump and Joe Biden, which social media platforms they use, and a few questions regarding their social media sharing preferences and tendencies (e.g., how important they think it is to share accurate news on social media).

**Question Wordings for Study 1**

*Party Affiliation*

Generally speaking, do you usually think of yourself as a Republican, a Democrat, or an Independent?

- Democrat
- Republican
- Independent

**PROGRAMING INSTRUCTION: IF ANSWERED DEMOCRAT OR REPUBLICAN, ASK, PUTTING IN THE APPROPRIATE PARTY:**

Would you call yourself a strong [**Democrat** / **Republican**] or a not very strong [**Democrat** / **Republican**]?

- Strong [**Democrat** / **Republican**]
- Not very strong [**Democrat** / **Republican**]

**PROGRAMING INSTRUCTION: IF ANSWERED INDEPENDENT, ASK:**

Do you think of yourself as closer to the Democratic or Republican Party?

- Closer to Democratic Party
- True Independent
- Closer to Republican Party

**PROGRAMING INSTRUCTION: IF ANSWERED TRUE INDEPENDENT, ASK:**

Thank you for answering. We understand that you consider yourself a true Independent. However, if forced to pick, which political party do you identify more closely with?

- Democratic Party
- Republican Party

*Headline Questions*

**[Participants loop through this block 10 times and, each time, are shown one randomly sampled headline from the set of 140 headlines used in study 1. All headlines are available on** [**OSF**](https://osf.io/5jwgd/?view_only=b8de674a8e9b4df5a5cb37a7c9fd84d3)**.]**

**Perceived accuracy**

What is the likelihood that the above headline is true?

- 1 - Not at all likely
- 2
- 3
- 4
- 5 - Somewhat likely
- 6
- 7
- 8
- 9 - Very likely

**Favorability to party**

Assuming the above headline is entirely accurate, how favorable would it be to Democrats versus Republicans?

- 1 - Very favorable for Democrats
- 2
- 3
- 4
- 5 - Neutral
- 6
- 7
- 8
- 9 - Very favorable for Republicans

**Benefit to party**

If you were to share the above headline on social media, how much would this benefit Democrats versus Republicans?

- 1 - Strongly benefits Democrats
- 2
- 3
- 4
- 5 - Neutral
- 6
- 7
- 8
- 9 - Strongly benefits Republicans

**Importance**

Assuming the headline is entirely accurate, how important would this news be?

- 1 - Not at all important
- 2
- 3
- 4
- 5 - Somewhat important
- 6
- 7
- 8
- 9 - Very important

**Funny**

How funny is this headline?

- 1 - Not at all funny
- 2
- 3
- 4
- 5 - Somewhat funny
- 6
- 7
- 8
- 9 - Very funny

**Surprising**

How surprising is this headline?

- 1 - Not at all surprising
- 2
- 3
- 4
- 5 - Somewhat surprising
- 6
- 7
- 8
- 9 - Very surprising

**Anticipated reputational gain**

If you were to share this headline on social media (such as Facebook, Twitter, or Instagram), using your own account…

How positively would you be viewed, overall, by people in your social network?

- 1 - Not at all
- 2
- 3
- 4
- 5 - Somewhat
- 6
- 7
- 8
- 9 - Very

**PROGRAMING INSTRUCTION: FILL IN WITH THE APPROPRIATE PARTY FROM THE PARTY AFFILIATION QUESTION:**

**Strong and loyal partisan**

If you were to share this headline on social media (such as Facebook, Twitter, or Instagram), using your own account…

How much would people in your social network see you as a strong and loyal [**Democrat** / **Republican**]?

- 1 - Not at all
- 2
- 3
- 4
- 5 - Somewhat
- 6
- 7
- 8
- 9 - Very

**Anticipated engagement**

If you were to share this headline on social media (such as Facebook, Twitter, or Instagram), using your own account…

How likely would people in your social network be to engage with your post (e.g., by liking it, commenting on it, or sharing it)?

- 1 - Not at all
- 2
- 3
- 4
- 5 - Somewhat
- 6
- 7
- 8
- 9 - Very

*Demographics and exploratory questions*

What is your age?

- [text entry box]

What is your gender?

- Male
- Female
- Transgender Female
- Transgender Male
- Trans/Non-Binary
- Not listed [text entry box]
- Prefer not to answer

What is the highest level of school you have completed or the highest degree you have received?

- High school graduate (high school diploma or equivalent including GED)
- Some college but no degree
- Associate degree in college (2-year)
- Bachelor's degree in college (4-year)
- Master's degree
- Doctoral degree
- Professional degree (JD, MD)

Information about income is very important to understand. Would you please give your best guess? Please indicate the answer that includes your entire household income in 2020 before taxes.

- Less than $10,000
- $10,000 to $19,999
- $20,000 to $29,999
- $30,000 to $39,999
- $40,000 to $49,999
- $50,000 to $59,999
- $60,000 to $69,999
- $70,000 to $79,999
- $80,000 to $89,999
- $90,000 to $99,999
- $100,000 to $149,999
- $150,000 or more

Please choose whichever ethnicity that you identify with (you may choose more than one option):

- White/Caucasian
- Asian
- Black or African American
- Native Hawaiian or Pacific Islander
- American Indian or Alaska Native
- Other [text entry box]

In general, how conservative or liberal do you consider yourself to be?

- 1 - Very conservative
- 2
- 3
- 4
- 5 - Neither liberal nor conservative
- 6
- 7
- 8
- 9 - Very liberal

On social issues, how conservative or liberal do you consider yourself to be?

- 1 - Very conservative
- 2
- 3
- 4
- 5 - Neither liberal nor conservative
- 6
- 7
- 8
- 9 - Very liberal

On fiscal issues, how conservative or liberal do you consider yourself to be?

- 1 - Very conservative
- 2
- 3
- 4
- 5 - Neither liberal nor conservative
- 6
- 7
- 8
- 9 - Very liberal

Now, please answer the following question not about yourself, but about the people you tend to be socially connected to.

In general, how conservative or liberal do these people tend to be?

- 1 - Very conservative
- 2
- 3
- 4
- 5 - Neither liberal nor conservative
- 6
- 7
- 8
- 9 - Very liberal

Who did you vote for in the 2020 Presidential Election?

Reminder: This survey is anonymous.

- Joseph Biden
- Donald Trump
- Other candidate
- I did not vote for reasons outside of my control
- I did not vote, but I could have
- I did not vote out of protest

Who did you vote for in the 2018 Congressional Election (if anyone)?

Reminder: This survey is anonymous.

- The Democratic Party candidate in my district
- The Republican Party candidate in my district
- Other candidate
- I did not vote for reasons outside of my control
- I did not vote, but I could have
- I did not vote out of protest

How favorable do you feel towards Donald Trump?

[Slider from 0 to 100 – Extremely cold (0) to No feeling (50) to Extremely warm (100)]

How favorable do you feel towards Joe Biden?

[Slider from 0 to 100 – Extremely cold (0) to No feeling (50) to Extremely warm (100)]

Do you support or oppose Donald Trump's presidency?

- (1) Strongly oppose
- 2
- 3
- (4) Neither oppose nor support
- 5
- 6
- (7) Strongly support

Please rate your agreement with the following statement:

Joe Biden legitimately won the 2020 U.S. Presidential Election.

- (1) Strongly disagree
- 2
- 3
- (4) Neither agree nor disagree
- 5
- 6
- (7) Strongly agree

Do you have a Facebook account?

- Yes
- No

Do you have a Twitter account?

- Yes
- No

How frequently do you use social media accounts (such as Facebook and Twitter)?

- Never
- Once a month
- Once a week
- 2-6 times a week
- Daily

Would you ever consider sharing something political on social media accounts (such as Facebook and Twitter)?

- Yes
- No
- I don’t use social media accounts

How important is it to you that you only share news articles on social media (such as Facebook and Twitter) if they are accurate?

- Not at all important
- Slightly important
- Moderately important
- Very important
- Extremely important

Did you respond randomly at any point during the study?

Note: Please be honest! You will get your payment regardless of your response.

- Yes
- No

Did you search the internet (via Google or otherwise) for any of the news headlines?

Note: Please be honest! You will get your HIT regardless of your response.

- Yes
- No

Thanks for taking our survey! We welcome your comments and feedback:

- [text entry box]

How long did the survey take you, approximately?

- [text entry box]

**Study 2 Procedure (preregistered)**

Study 2 was preregistered and was conducted in November 2021. Participants provided informed consent and were then given the same first attention check from Study 1. As was done in Study 1, participants who did not pass the first attention check were not allowed to complete the survey and we only analyzed data from participants who passed both attention checks.

Participants were then told that they would be presented with a series of actual news headlines and that they would answer a series of questions for each headline. Participants were then shown 10 headlines that were randomly sampled from a total set of 200 headlines. 30 of these headlines came from the 140 headlines from Study 1 but the rest were all unique to Study 2. Each headline was shown one at a time and participants were asked questions related to one headline before being shown the next headline. Included in these questions were our main independent variables (political favorability and perceived accuracy; both preregistered) and our main dependent variable (anticipated reputational gain; preregistered).

First, participants were asked to rate the political favorability of the headline using the same question from Study 1. However, Studies 2 and 3 used a 6-pt scale (1 = more favorable for Democrats; 6 = more favorable for Republicans) for this question. As was done in Study 1, this question was recoded in Studies 2 and 3 with respect to the participant’s party such that a higher score means higher favorability to the participant’s own party (i.e., reverse coded for Democrats).

Participants were then asked to rate the perceived accuracy of the headline using the same question from Study 1. However, Studies 2 and 3 used a 6-pt scale (1 = extremely unlikely; 6 = extremely likely) for this question.

Participants were then asked the following four questions, presented in a random order: how important, boring, positive, and negative the headline is.

To rate how important the headline is, participants were asked: “Assuming the headline is entirely accurate, how important would this news be?” Participants used a 6-pt scale (1 = extremely unimportant; 6 = extremely important) to answer this question.

To rate how boring the headline is, participants were asked: “How boring is this headline?” Participants used a 6-pt scale (1 = not at all; 6 = extremely) to answer this question.

To rate how positive the headline is, participants were asked: “To what extent does this headline make you feel good (happy, excited, hopeful, etc.)?” Participants used a 6-pt scale (1 = not at all; 6 = extremely) to answer this question.

To rate how negative the headline is, participants were asked: “To what extent does this headline make you feel bad (angry, sad, worried, etc.)?” Participants used a 6-pt scale (1 = not at all; 6 = extremely) to answer this question.

Participants were then asked how familiar they were with the headline: “Are you familiar with the above headline (have you seen or heard about it before)?” Participants used a 6-pt scale (1 = not at all; 6 = extremely) to answer this question.

Participants were then asked how likely it is that they would share the headline on social media: “If you were to see the above article on social media, how likely would you be to share it?” Participants used a 6-pt scale (1 = extremely unlikely; 6 = extremely likely) to answer this question.

Participants then answered our key dependent variable, anticipated reputational gain, which was measured using the following question: “Imagine that you shared the above article on social media. How would you be viewed by people in your social network?” For Studies 2 and 3, participants used a 7-pt scale to answer this question (1 = Extremely negatively; 4 (midpoint) = neither negatively nor positively; 7 = Extremely positively).

After answering the questions for all ten headlines, participants were then given the same second attention check from Study 1. After the second attention check, participants then completed two additional measures: the Actively Open-minded (AOT) scale and the Willingness to Self-Censor Scale (WTSCS).

Participants were then asked if they were a Democrat, Republican, independent, or “other”. They were also asked to indicate if they were a strong Democrat or Republican. For Study 2, we preregistered that we would restrict our analyses to participants who identify as either a Democrat or Republican (rather than an independent or “other”).

Finally, participants were asked the following demographic and exploratory questions: political ideology, age, gender, education, income, ethnicity, belief in God, whether they responded randomly during the study, and whether they used Google during the study to look up any headlines.

**Question Wordings for Study 2**

*Headline Questions*

**[Participants loop through this block 10 times and, each time, are shown one randomly sampled headline from the set of 200 headlines used in study 2. All headlines are available on** [**OSF**](https://osf.io/5jwgd/?view_only=b8de674a8e9b4df5a5cb37a7c9fd84d3)**.]**

**Favorability to party**

Assuming the above headline is entirely accurate, how favorable would it be to Democrats versus Republicans?

- More favorable for Democrats
- Moderately more favorable for Democrats
- Slightly more favorable for Democrats
- Slightly more favorable for Republicans
- Moderately more favorable for Republicans
- More favorable for Republicans

**Perceived accuracy**

What is the likelihood that the above headline is true?

- Extremely unlikely
- Moderately unlikely
- Slightly unlikely
- Slightly likely
- Moderately likely
- Extremely likely

**Importance**

Assuming the headline is entirely accurate, how important would this news be?

- Extremely unimportant
- Moderately unimportant
- Slightly unimportant
- Slightly important
- Moderately important
- Extremely important

**Boring**

How boring is this headline?

- Not at all
- Slightly
- Somewhat
- Moderately
- Very much
- Extremely

**Positive**

To what extent does this headline make you feel good (happy, excited, hopeful, etc.)?

- Not at all
- Slightly
- Somewhat
- Moderately
- Very much
- Extremely

**Negative**

To what extent does this headline make you feel bad (angry, sad, worried, etc.)?

- Not at all
- Slightly
- Somewhat
- Moderately
- Very much
- Extremely

**Familiarity**

Are you familiar with the above headline (have you seen or heard about it before)?

- Not at all
- Slightly
- Somewhat
- Moderately
- Very much
- Extremely

**Share**

If you were to see the above article on social media, how likely would you be to share it?

- Extremely unlikely
- Moderately unlikely
- Slightly unlikely
- Slightly likely
- Moderately likely
- Extremely likely

**Anticipated reputational gain**

Imagine that you shared the above article on social media. How would you be viewed by people in your social network?

- Extremely negatively
- Moderately negatively
- Slightly negatively
- Neither negatively or positively
- Slightly positively
- Moderately positively
- Extremely positively

*Actively Open-minded (AOT) scale*

It is important to be loyal to your beliefs even when evidence is brought to bear against them.

- Strongly disagree
- Disagree
- Somewhat disagree
- Somewhat agree
- Agree
- Strongly agree

Whether something feels true is more important than evidence.

- Strongly disagree
- Disagree
- Somewhat disagree
- Somewhat agree
- Agree
- Strongly agree

Just because evidence conflicts with my current beliefs does not mean my beliefs are wrong.

- Strongly disagree
- Disagree
- Somewhat disagree
- Somewhat agree
- Agree
- Strongly agree

There may be evidence that goes against what you believe but that does not mean you have to change your beliefs.

- Strongly disagree
- Disagree
- Somewhat disagree
- Somewhat agree
- Agree
- Strongly agree

Even if there is concrete evidence against what you believe to be true, it is OK to maintain cherished belief.

- Strongly disagree
- Disagree
- Somewhat disagree
- Somewhat agree
- Agree
- Strongly agree

Regardless of the topic, what you believe to be true is more important than evidence against your beliefs.

- Strongly disagree
- Disagree
- Somewhat disagree
- Somewhat agree
- Agree
- Strongly agree

*Willingness to Self-Censor Scale (WTSCS)*

It is difficult for me to express my opinion if I think others won't agree with what I say.

- Strongly disagree
- Disagree
- Neither agree nor disagree
- Agree
- Strongly agree

There have been many times when I have thought others around me were wrong but I didn't let them know.

- Strongly disagree
- Disagree
- Neither agree nor disagree
- Agree
- Strongly agree

When I disagree with others, I'd rather go along with them than argue about it.

- Strongly disagree
- Disagree
- Neither agree nor disagree
- Agree
- Strongly agree

It is easy for me to express my opinion around others who I think will disagree with me.

- Strongly disagree
- Disagree
- Neither agree nor disagree
- Agree
- Strongly agree

I'd feel uncomfortable if someone asked my opinion and I knew that he or she wouldn't agree with me.

- Strongly disagree
- Disagree
- Neither agree nor disagree
- Agree
- Strongly agree

I tend to speak my opinion only around friends or other people I trust.

- Strongly disagree
- Disagree
- Neither agree nor disagree
- Agree
- Strongly agree

It is safer to keep quiet than publicly speak an opinion that you know most others don't share.

- Strongly disagree
- Disagree
- Neither agree nor disagree
- Agree
- Strongly agree

If I disagree with others, I have no problem letting them know it.

- Strongly disagree
- Disagree
- Neither agree nor disagree
- Agree
- Strongly agree

*Politics*

Which of the following best describes your political position?

- Democrat
- Republican
- Independent
- Other (specify) [text entry box]

Which of the following best describes your political preference?

- Strongly Democratic
- Democratic
- Lean Democratic
- Lean Republican
- Republican
- Strongly Republican

On social issues I am:

- Strongly Liberal
- Somewhat Liberal
- Moderate
- Somewhat Conservative
- Strongly Conservative

On economic issues I am:

- Strongly Liberal
- Somewhat Liberal
- Moderate
- Somewhat Conservative
- Strongly Conservative

*Demographics*

What is your age?

- [text entry box]

What is your gender?

- Male
- Female
- Transgender Female
- Transgender Male
- Trans/Non-Binary
- Not listed [text entry box]
- Prefer not to answer

What is the highest level of school you have completed or the highest degree you have received?

- High school graduate (high school diploma or equivalent including GED)
- Some college but no degree
- Associate degree in college (2-year)
- Bachelor's degree in college (4-year)
- Master's degree
- Doctoral degree
- Professional degree (JD, MD)

Information about income is very important to understand. Would you please give your best guess? Please indicate the answer that includes your entire household income in 2020 before taxes.

- Less than $10,000
- $10,000 to $19,999
- $20,000 to $29,999
- $30,000 to $39,999
- $40,000 to $49,999
- $50,000 to $59,999
- $60,000 to $69,999
- $70,000 to $79,999
- $80,000 to $89,999
- $90,000 to $99,999
- $100,000 to $149,999
- $150,000 or more

Please choose whichever ethnicity that you identify with (you may choose more than one option):

- White/Caucasian
- Asian
- Black or African American
- Native Hawaiian or Pacific Islander
- American Indian or Alaska Native
- Other [text entry box]

How much would you say you believe in God or Gods?

- Not at all 0
- 1
- 2
- 3
- 4
- 5
- 6
- Very much 7

Did you respond randomly at any point during the study?

Note: Please be honest! You will get your payment regardless of your response.

- Yes
- No

Did you search the internet (via Google or otherwise) for any of the news headlines?

Note: Please be honest! You will get your HIT regardless of your response.

- Yes
- No

Thanks for taking our survey! We welcome your comments and feedback:

- [text entry box]

How long did the survey take you, approximately?

- [text entry box]

**Study 3 Procedure (preregistered)**

Study 3 was preregistered and was conducted in April 2022. Participants provided informed consent and were then given the same first attention check from Studies 1 and 2. As was done in Studies 1 and 2, participants who did not pass the first attention check were not allowed to complete the survey and we only analyzed data from participants who passed both attention checks.

Participants were then told that they would be presented with a series of actual news headlines and that they would answer a series of questions for each headline. Participants were then shown 10 headlines that were randomly sampled from a total set of 278 headlines. Each headline was unique to Study 3 (i.e., no headlines came from either Studies 1 or 2). Each headline was shown one at a time and participants were asked questions related to one headline before being shown the next headline. Included in these questions were our main independent variables (political favorability and perceived accuracy; both preregistered) and our main dependent variable (anticipated reputational gain; preregistered). All questions were presented in a random order except the final two questions asking participants how likely they would be to share the headline on social media and our main dependent variable, anticipated reputational gain.

Participants were asked about the political favorability as well as the perceived accuracy of the headline, following the same procedure from Study 2.

Participants were also asked how sensational the headline is: “To what extent is this headline sensationalist and/or exaggerated?” Participants used a 6-pt scale (1 = not at all; 6 = extremely) to answer this question.

Participants were asked how informative the headline is: “How informative is this headline?” Participants used a 6-pt scale (1 = not at all; 6 = extremely) to answer this question.

Participants were asked how surprising the headline is: “How surprising is this headline?” Participants used a 6-pt scale (1 = not at all; 6 = extremely) to answer this question.

Participants were asked how impactful/important the headline is: “How impactful/important is this headline?” Participants used a 6-pt scale (1 = not at all; 6 = extremely) to answer this question.

Participants were then asked how familiar they were with the headline: “Are you familiar with the above headline (have you seen or heard about it before)?” Participants used a 6-pt scale (1 = not at all; 6 = extremely) to answer this question.

Participants were then asked how likely it is that they would share the headline on social media: “If you were to see the above article on social media, how likely would you be to share it?” Participants used a 6-pt scale (1 = extremely unlikely; 6 = extremely likely) to answer this question.

Finally, participants answered our key dependent variable, anticipated reputational gain, following the same procedure from Study 2.

After answering the questions for all ten headlines, participants were then given the same second attention check from Study 1 and 2. Participants were then asked if they were a Democrat, Republican, independent, or “other”. They were also asked to indicate if they were a strong Democrat or Republican. As was done in Study 2, we preregistered Study 3 such that we would restrict our analyses to participants who identify as either a Democrat or Republican (rather than an independent or “other”).

Finally, participants were asked the following demographic and exploratory questions: political ideology, age, gender, education, income, ethnicity, belief in God, whether they responded randomly during the study, and whether they used Google during the study to look up any headlines.

**Question Wordings for Study 3**

*Headline Questions*

**[Participants loop through this block 10 times and, each time, are shown one randomly sampled headline from the set of 278 headlines used in study 3. All headlines are available on** [**OSF**](https://osf.io/5jwgd/?view_only=b8de674a8e9b4df5a5cb37a7c9fd84d3)**.]**

**Favorability to party**

Assuming the above headline is entirely accurate, how favorable would it be to Democrats versus Republicans?

- More favorable for Democrats
- Moderately more favorable for Democrats
- Slightly more favorable for Democrats
- Slightly more favorable for Republicans
- Moderately more favorable for Republicans
- More favorable for Republicans

**Perceived accuracy**

What is the likelihood that the above headline is true?

- Extremely unlikely
- Moderately unlikely
- Slightly unlikely
- Slightly likely
- Moderately likely
- Extremely likely

**Sensational**

How sensational is this headline?

- Not at all
- Slightly
- Somewhat
- Moderately
- Very much
- Extremely

**Informative**

How informative is this headline?

- Not at all
- Slightly
- Somewhat
- Moderately
- Very much
- Extremely

**Surprising**

How surprising is this headline?

- Not at all
- Slightly
- Somewhat
- Moderately
- Very much
- Extremely

**Importance**

How impactful/important is this headline?

- Not at all
- Slightly
- Somewhat
- Moderately
- Very much
- Extremely

**Familiarity**

Are you familiar with the above headline (have you seen or heard about it before)?

- Not at all
- Slightly
- Somewhat
- Moderately
- Very much
- Extremely

**Share**

If you were to see the above article on social media, how likely would you be to share it?

- Extremely unlikely
- Moderately unlikely
- Slightly unlikely
- Slightly likely
- Moderately likely
- Extremely likely

**Anticipated reputational gain**

Imagine that you shared the above article on social media. How would you be viewed by people in your social network?

- Extremely negatively
- Moderately negatively
- Slightly negatively
- Neither negatively or positively
- Slightly positively
- Moderately positively
- Extremely positively

*Politics*

Which of the following best describes your political position?

- Democrat
- Republican
- Independent
- Other (specify) [text entry box]

Which of the following best describes your political preference?

- Strongly Democratic
- Democratic
- Lean Democratic
- Lean Republican
- Republican
- Strongly Republican

On social issues I am:

- Strongly Liberal
- Somewhat Liberal
- Moderate
- Somewhat Conservative
- Strongly Conservative

On economic issues I am:

- Strongly Liberal
- Somewhat Liberal
- Moderate
- Somewhat Conservative
- Strongly Conservative

*Demographics*

What is your age?

- [text entry box]

What is your gender?

- Male
- Female
- Transgender Female
- Transgender Male
- Trans/Non-Binary
- Not listed [text entry box]
- Prefer not to answer

What is the highest level of school you have completed or the highest degree you have received?

- High school graduate (high school diploma or equivalent including GED)
- Some college but no degree
- Associate degree in college (2-year)
- Bachelor's degree in college (4-year)
- Master's degree
- Doctoral degree
- Professional degree (JD, MD)

Information about income is very important to understand. Would you please give your best guess? Please indicate the answer that includes your entire household income in 2020 before taxes.

- Less than $10,000
- $10,000 to $19,999
- $20,000 to $29,999
- $30,000 to $39,999
- $40,000 to $49,999
- $50,000 to $59,999
- $60,000 to $69,999
- $70,000 to $79,999
- $80,000 to $89,999
- $90,000 to $99,999
- $100,000 to $149,999
- $150,000 or more

Please choose whichever ethnicity that you identify with (you may choose more than one option):

- White/Caucasian
- Asian
- Black or African American
- Native Hawaiian or Pacific Islander
- American Indian or Alaska Native
- Other [text entry box]

How much would you say you believe in God or Gods?

- Not at all 0
- 1
- 2
- 3
- 4
- 5
- 6
- Very much 7

Did you respond randomly at any point during the study?

Note: Please be honest! You will get your payment regardless of your response.

- Yes
- No

Did you search the internet (via Google or otherwise) for any of the news headlines?

Note: Please be honest! You will get your HIT regardless of your response.

- Yes
- No

Thanks for taking our survey! We welcome your comments and feedback:

- [text entry box]

How long did the survey take you, approximately?

- [text entry box]

**Additional information**

For all studies, we excluded responses from participants who did not pass both attention checks.

We also excluded responses from participants who did not answer questions for all ten headlines. Additionally, for any duplicated responses that came from the same IP address, we only included the chronologically first response. All of these exclusion criteria were preregistered for Study 2 and 3.

For the survey analyses presented in the main text, we aimed to have a more objective measure of the political favorability of each headline in addition to participants’ perceived accuracy ratings. To obtain this, we included a binary measure of veracity for each headline, which categorized headlines as either “false” or “true” based on where the headline was obtained from. Specifically, we obtained all of our “false” headlines from fact-checking outlets (e.g., Snopes) that classified the headline as false; we obtained all of our “true” headlines from reputable mainstream sources (e.g., NPR). Although all of our headlines either came from fact-checking outlets that labeled the headline as false or from reputable mainstream sources, the inclusion of this binary measure was not preregistered.

Finally, our main DV, anticipated reputational gain, was measured using a different scale range across the three studies: a 9-pt scale in Study 1 and a 7-pt scale in Studies 2 and 3. For ease of interpretation, we rescaled this DV to be on a 0-1 scale when plotting the results in the main text.

**Description of Samples**

Participants in Study 1 were recruited from Amazon Mechanical Turk without any stratified sampling procedure (i.e., randomly sampled). Participants in Studies 2 and 3 were recruited from Lucid and were quota-matched to the national distribution on age, gender, ethnicity, and geographic region. Participants in all studies were only included in our analyses if they passed our attention checks (described in the procedures for Studies 1-3 in the Extended Methods).

Below is a demographic breakdown of the sample for Study 1:

The mean age of participants was 41.89 years with a standard deviation of 12.92 years. The gender, race/ethnicity (for which respondents could choose more than one category), and educational breakdown of the sample is as follows: Male (37.00%), Female (61.49%), Transgender (1.12%), Other (<1%); White/Caucasian (86.05%), Black or African American (6.70%), American Indian or Alaska Native (1.44%), Asian (5.61%), Native Hawaiian or Pacific Islander (0.61%), Other (2.43%); Less than high school degree (0.23%), High school graduate (high school diploma or equivalent including GED) (7.74%), Some college (20.26%), Associate degree in college (10.70%), Bachelor’s degree in college (41.20%), Advanced degree (19.88%). With respect to political affiliation: Democrat (41.24%), Republican (49.43%), and Independent (9.33%).

Below is a demographic breakdown of the sample for Study 2:

The mean age of participants was 47.21 years with a standard deviation of 17.78 years. The gender, race/ethnicity (for which respondents could choose more than one category), and educational breakdown of the sample is as follows: Male (46.98%), Female (51.92%), Transgender (<1%), Other (<1%); White/Caucasian (80.30%), Black or African American (11.45%), American Indian or Alaska Native (2.88%), Asian (4.60%), Native Hawaiian or Pacific Islander (0.55%), Other (4.36%); Less than high school degree (3.29%), High school graduate (high school diploma or equivalent including GED) (27.41%), Some college (23.10%), Associate degree in college (11.20%), Bachelor’s degree in college (24.04%), Advanced degree (10.97%). With respect to political affiliation: Democrat (36.76%), Republican (30.09%), Independent (29.23%), and Other (3.92%).

Below is a demographic breakdown of the sample for Study 3:

The mean age of participants was 47.48 years with a standard deviation of 17.11 years. The gender, race/ethnicity (for which respondents could choose more than one category), and educational breakdown of the sample is as follows: Male (47.98%), Female (50.83%), Transgender (<1%), Other (<1%); White/Caucasian (80.65%), Black or African American (10.51%), American Indian or Alaska Native (2.13%), Asian (5.13%), Native Hawaiian or Pacific Islander (0.71%), Other (3.79%); Less than high school degree (3.16%), High school graduate (high school diploma or equivalent including GED) (23.62%), Some college (23.38%), Associate degree in college (12.95%), Bachelor’s degree in college (21.41%), Advanced degree (15.48%). With respect to political affiliation: Democrat (38.40%), Republican (30.17%), Independent (27.95%), and Other (3.48%).

**Extended Methods for the Twitter analysis**

**Data Set Construction**

The details of the Twitter data set construction as well as the creation of the dependent variables were all preregistered. We were able to retrieve the original URL for 502 of the 588 unique news headlines used across the three surveys (we say “unique” since some headlines appeared in more than one survey). To create the data set used in the Twitter analyses, we took the URL for these headlines and used the Twitter Academic API to identify all Twitter users who had posted primary tweets containing each URL. These primary tweets occurred between 2016 and 2022. This left us with 378 headlines that had at least one tweet containing that headline URL. Prior to preregistering analyses with the Twitter dataset, we ran some exploratory analyses with a small subset of the headlines (N = 43 headlines). Per our preregistered analysis plan, we excluded these 43 headlines when conducting the analyses reported in the main text, which left us with N = 335 headlines with corresponding Twitter approval data. For each news headline, we collected the total number of "likes" as well as the total number of "retweets'' that all tweets containing the link for that headline received.

For all tweets that we collected, we estimated the ideological preference of the Twitter user who authored the tweet, following the procedure of Barbera et al., 2015. This procedure employs a network-sampling technique to identify the latent ideological preference of Twitter users, operating under the assumption that individuals with similar political ideologies are more likely to follow each other on Twitter compared to those with differing political ideologies. As in Barbera et al., 2015, we set a value of 0.5 in the latent ideological dimension as the threshold to label Twitter users as "Republicans" and "Democrats" (>.5 for "Republicans" and <-.5 for "Democrats").

We then classified each news headline as “Democrat leaning” or “Republican leaning” using the political favorability ratings from our surveys. Due to the difference in scales between the surveys, we rescaled these values to range from 0 to 1. For the purposes of classifying headlines, we coded the political favorability ratings such that higher scores always indicate greater perceived favorability to Democrats. Then, headlines were classified as “Democrat leaning” if they had a value of 0.5 (the midpoint) or higher, while headlines that had a value of less than 0.5 were classified as “Republican leaning”.

Lastly, we chose to constrain our analyses to only include tweets with headlines whose partisan lean “matched” the estimated partisanship of the Twitter user who authored the tweet. We made this decision because we worried that, when users tweeted politically-incongruent headlines and received social approval from doing so, their tweets might reflect criticism (rather than endorsement) of the relevant headline, complicating the interpretation of the associations between headline attributes (i.e., veracity and political favorability) and social approval.

As a result, some headlines were excluded from analysis since they did not have any tweets by users whose partisan lean matched that of the headline. For example, if a headline was classified as "Democrat leaning" but there were no tweets authored by Democrat-identified users for that headline, we excluded this headline from our main analysis, even if there were tweets for this headline in general. This left us with a final N = 282 headlines (drawing on data from 26,154 tweets by authors whose partisan lean “matched” the headline). Then, within this set of headlines, our analyses allowed us to investigate variability in *how* favorable a headline was to the user’s party (from neutral to extremely favorable).

**Variable Construction**

To compute the veracity and political favorability metrics for each headline, we utilized average ratings from the surveys. Most of the headlines appeared in only one survey. However, 30 headlines overlapped between Study 1 and 2. For each of these 30 headlines, we computed the average rating within each survey, and then averaged the two resulting averages (using a weighted average based on the number of participants who rated the headline for each study). Given the different scales across the three surveys, we normalized veracity and favorability values to range from 0 to 1. Critically, we only used ratings from participants whose partisanship matched the partisan lean of the headline. For instance, a “Republican leaning” headline’s veracity and favorability scores were computed by averaging Republican participants’ ratings. This decision was made to emulate the social network environment of Twitter users, thereby simulating what their network members would likely perceive as accurate and politically favorable. Moreover, since favorability was coded in our surveys to indicate favorability to the participant’s *own* political party, by averaging across only Democrat or Republican ratings for each headline, favorability can be interpreted as the political favorability of the headline to the Twitter user’s (predicted) party (since partisan lean of the headline and the user is always matched).

Finally, we followed the procedure from Frimer et al. (2023) to compute the logged likes-per-retweet metric for each headline. Specifically, for any headlines that had zero total likes or zero total retweets, we first converted these zeros to 0.1s. We then divided total likes by total retweets and performed a log10 transformation on these ratios. We chose this metric as our measure of social approval in order to account for confounding factors such as follower count, which significantly influence post engagement metrics. Because of the potential confounding effects of follower count and other extraneous variables, we chose not to simply examine the total engagement (e.g., average like or retweet count) that tweets received. Instead, we employed the logged likes-to-retweets ratio as a measure of social approval, a metric validated in previous research (Frimer et al., 2023). Frimer et al. (2023) demonstrated that while absolute likes and retweets are confounded by variables such as follower count, the logged likes-per-retweet ratio correlates positively with two measures of social approval: positive sentiment in tweet replies and social approval as assessed by participants in separate surveys.

The rationale for using this ratio is that factors like follower count influence both the number of likes (numerator) and the number of retweets (denominator). By dividing likes by retweets and then logging the result, the ratio normalizes the influence of follower count, providing a more accurate measure of social approval. This metric captures the proportion of instances in which users, upon viewing a tweet in their feed, responded positively enough to "like" it. Consequently, we did not control for follower count in our analyses, as the logged likes-per-retweet metric already accounts for this variable.

**SI References**

Barberá, P. (2015). Birds of the same feather tweet together: Bayesian ideal point estimation using Twitter data. *Political analysis*, *23*(1), 76-91.

Frimer, J. A., Aujla, H., Feinberg, M., Skitka, L. J., Aquino, K., Eichstaedt, J. C., & Willer, R. (2023). Incivility is rising among American politicians on Twitter. *Social Psychological and Personality Science*, *14*(2), 259-269.
